# Cardiologist and cardiac surgeon view on decision-making in prosthetic aortic valve selection: does profession matter?

**DOI:** 10.1007/s12471-014-0564-6

**Published:** 2014-06-12

**Authors:** N. M. Korteland, J. Kluin, R. J. M. Klautz, J. W. Roos-Hesselink, M. I. M. Versteegh, A. J. J. C. Bogers, J. J. M. Takkenberg

**Affiliations:** 1 Department of Cardiothoracic Surgery, Erasmus MC, Bd563, PO Box 2040, 3000CA Rotterdam, the Netherlands; 2 Department of Cardiology, Erasmus MC, Rotterdam, the Netherlands; 3 Department of Cardiothoracic Surgery, UMC Utrecht, Utrecht, the Netherlands; 4 Department of Cardiothoracic Surgery, LUMC, Leiden, the Netherlands

**Keywords:** Shared decision-making, Aortic valve replacement, Aortic valve prostheses

## Abstract

**Aims:**

Assess and compare among Dutch cardiothoracic surgeons and cardiologists: opinion on (1) patient involvement, (2) conveying risk in aortic valve selection, and (3) aortic valve preferences.

**Methods and results:**

A survey among 117 cardiothoracic surgeons and cardiologists was conducted. Group responses were compared using the Mann–Whitney U test. Most respondents agreed that patients should be involved in decision-making, with surgeons leaning more toward patient involvement (always: 83 % versus 50 % respectively; *p* < 0.01) than cardiologists. Most respondents found that ideally doctors and patients should decide together, with cardiologists leaning more toward taking the lead compared with surgeons (*p* < 0.01).

Major risks of the therapeutic options were usually discussed with patients, and less common complications to a lesser extent. A wide variation in valve preference was noted with cardiologists leaning more toward mechanical prostheses, while surgeons more often preferred bioprostheses (*p* < 0.05).

**Conclusion:**

Patient involvement and conveying risk in aortic valve selection is considered important by cardiologists and cardiothoracic surgeons. The medical profession influences attitude with regard to aortic valve selection and patient involvement, and preference for a valve substitute. The variation in valve preference suggests that in most patients both valve types are suitable and aortic valve selection may benefit from evidence-based informed shared decision-making.

## Introduction

For most patients with severe aortic valve disease, aortic valve replacement is the treatment of choice. For the majority of patients two options exist: mechanical or bioprosthetic aortic valve replacement [1]. The decision for a particular prosthetic valve type is ideally driven by scientific evidence on patient outcome after implantation with different valve substitutes, the patient’s clinical state and circumstances, and informed patient preferences. Each valve type has specific advantages and disadvantages. Mechanical valves are designed to last a lifetime, so a lower re-operation hazard can be anticipated, compared with bioprosthetic valves. However, mechanical valves carry an increased thrombotic risk and therefore require lifelong anticoagulation [2]. Clinical characteristics such as age, anticipated life expectancy, indication/contraindication for anticoagulation, and comorbidities play an important role in the decision-making process [3]. Given the different nature of the pros and cons of different prosthetic valves, informed patient preferences deserve consideration in the decision-making. Shared decision-making is receiving more and more attention in healthcare [4]. Using shared decision-making, patients are stimulated to think about their treatment, about treatment options and associated benefits and harm so they can place these in their own personal context and discuss their preferences with the physician and then decide with their physician what treatment option is best for them [5].

The 2012 ESC/EACTS Valvular Heart Disease Guidelines state that a mechanical or bioprosthetic valve should be recommended according to the desire of the informed patient [2]. But how do we inform the patient? And how do we assess patient preferences? The opinion of Dutch cardiothoracic surgeons and cardiologists on shared decision-making is as yet undefined [6]. To investigate shared decision-making in daily cardiovascular practice, we performed this study. The purpose of this study was to assess the expert opinion of the cardiothoracic surgeon and cardiologist on patient involvement and conveying risk in aortic valve selection, and to assess prosthetic aortic valve preferences of cardiac surgeons and cardiologists.

## Methods

A survey was administered to cardiothoracic surgeons (in training) during the semi-annual meeting of the Netherlands Association for Cardiothoracic Surgery (November 2011) and distributed among cardiothoracic surgeons in several institutions (2012). The same survey was administered to cardiologists (in training) attending the semi-annual meeting of the Netherlands Society of Cardiology (May 2012) and distributed among cardiologists in several institutions (2012).

The questionnaire consisted of five general questions: physician age, speciality (surgeon (in training), cardiologist (in training)), hospital, number of years in practice, and annual number of aortic valve replacements in their institution. The physicians were asked five questions to assess their opinion on involvement of patients in decision-making using a Control Preference scale [7, 8] and 5-point Likert scales [6, 9]. Physician perspective on discussing risks and benefits of different prosthetic valve types was assessed by rating how often each complication will be discussed using 5-point Likert scales ranging from never to always. Physician opinion on choice of treatment strategies was assessed by six hypothetical cases in which the physician rated the likelihood of choosing a particular prosthetic valve type using 7-point Likert scales ranging from 1 (definitely mechanical valve) to 7 (definitely bioprosthetic valve). For a detailed description of the questionnaire, see Appendix.

### Statistical methods

Continuous variables are displayed as mean, standard deviation and range, discrete variables as counts or proportions. Comparison of group characteristics was done using the unpaired t-test. Group responses are displayed as median, interquartile range, and total range. To compare group responses between surgeons and cardiologists and influence of physician age and cardiac surgery program in the respondent’s institution on survey response, the Mann–Whitney U-test was used at a probability value of 0.05. All tests were two-sided, and a p-value of 0.05 or less was considered statistically significant. All statistical analyses were performed using IBM-SPSS 20 (IBM Corp., Armonk, NY).

## Results

A total of 117 Dutch medical specialists from 38 different institutions participated. Mean age was 47 ± 10 years (range 26–67), mean clinical experience 14 ± 9 years (range 0–36). Fifty-four cardiothoracic surgeons (11 in training) represent 38 % of the Dutch cardiothoracic surgeon population, 63 cardiologists (7 in training) represent 6 % of the Dutch cardiologist population. There were no differences in age and clinical experience between the cardiothoracic surgeons and cardiologists.

### Physician view on patient participation in decision-making

**Fig. 1:**
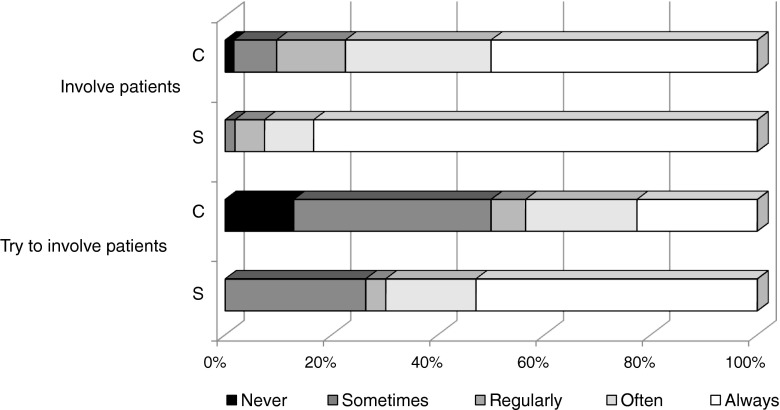
Cardiologist (C) and surgeon (S) preference for patient involvement and risk conveyance in aortic valve selection. Total *n* = 117, cardiologists = 63 and surgeons = 54. Difference between groups: *p < 0.01. *Pts* = patients. *QoL* = quality of life

**Fig. 2:**
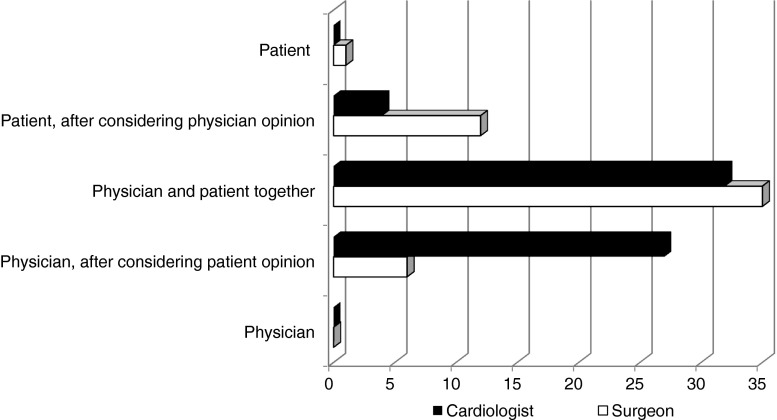
Physician preference for final decision in prosthetic aortic valve choice. Total *n* = 117, cardiologists = 63 and surgeons = 54. Difference between cardiologists and surgeons: *p* < 0.01

Figure 1 displays physician preferences for patient involvement and the conveying risk. Figure 2 displays physician preferences for final decision-making in prosthetic aortic valve choice. Subgroup analysis revealed that physicians above the age of 50 more often lean toward patient involvement in decision-making than physicians under age 50. Physicians working in a centre with cardiac surgery were more inclined to decide together with the patient, while physicians working in a centre without a cardiac surgery program more often preferred to take the lead in decision-making.

### Physician view on conveying risk and benefit

**Fig. 3:**
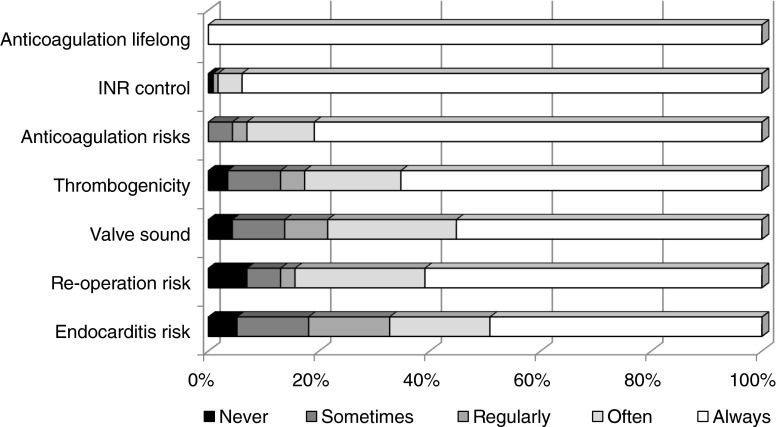
Physician responses regarding risk and benefit conveyance to patients about mechanical valves. Total *n* = 117

**Fig. 4:**
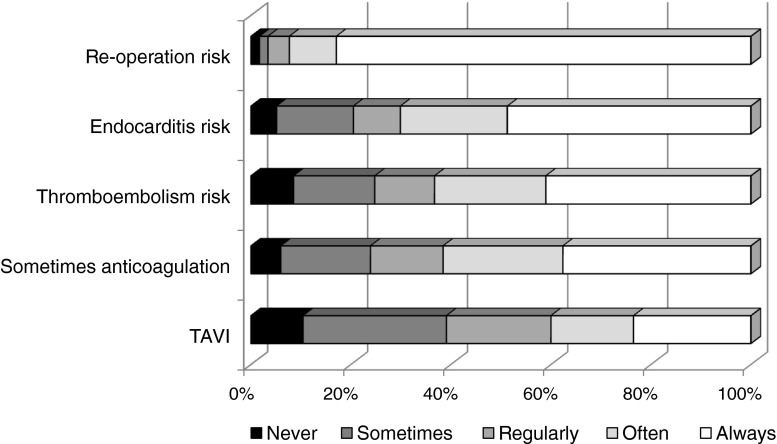
Physician responses regarding risk and benefit conveyance to patients about bioprosthetic valves. Total *n* = 117

Figures 3 and 4 summarise physician responses regarding conveying risk and benefit to patients about mechanical valves (Fig. 3) and bioprosthetic valves (Fig. 4).

There were no differences between surgeons and cardiologists regarding conveying risk and benefit to patients. Physicians under the age of 50 more often informed patients regarding anticoagulation risks than physicians older than age 50. Physicians working in a centre with cardiac surgery more often informed patients about the risks and benefits of a mechanical valve compared with those working in a centre without a cardiac surgery program.

### Physician prosthetic valve preferences

**Fig. 5:**
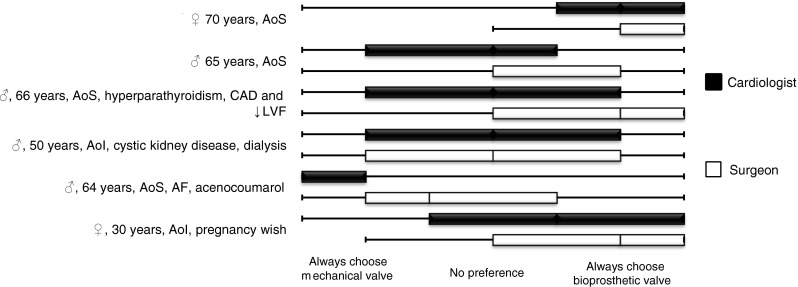
Physician preferences for a prosthetic valve type in 6 cases. The box contains 50 % of the physician preferences. The vertical line in the box represents the median, and the whiskers illustrate the minimum and maximum value. Total *n* = 117, cardiologists = 63 and surgeons = 54. Difference between groups: *p<0.05

The results of the answers to the six hypothetical patient cases are illustrated in the box-and-whisker plot in Fig. 5. Physicians above the age of 50 were leaning more toward mechanical valves compared with physicians under age 50. The presence of a cardiac surgery program in the respondent’s institution was not associated with prosthetic valve preferences in any of the six hypothetical cases.

## Discussion

Prosthetic aortic valve selection is a delicate process. By carefully considering research evidence on outcome after aortic valve replacement with different aortic valve prostheses, clinical characteristics and medical circumstances of the patient, and by taking patient preferences into account, the likelihood of making an optimal choice increases [1]. The results from this survey among Dutch cardiothoracic surgeons and cardiologists suggest that patient involvement in prosthetic aortic valve selection is considered important by most physicians. This finding is in line with previous research in other medical professions, showing that patients and physicians prefer the decision to be the outcome of a shared decision-making process [8, 10]. Dutch cardiothoracic surgeons lean more toward patient involvement than cardiologists, who prefer to take the lead in decision-making, as do physicians above the age of 50 and physicians who are working in an institution with a cardiac surgery program. These differences could be due to external factors in medical practice, such as differences in consulting time, and cultural differences. Since shared decision-making has not yet been standardised in clinical practice, there is probably a wide variation in the application of the concept in current clinical practice. Current practice in one hospital could, for example, be that the surgeons are mainly discussing the choice for a certain valve type with the patients, while in another clinic cardiologists do so, or both specialities together. Although the 2012 ESC/EACTS Valvular Heart Disease Guidelines state that a prosthetic valve should be recommended according to the desire of the informed patient [1], the way Dutch hospitals are following these guidelines probably differs a lot. The Netherlands Association for Cardiothoracic Surgery and the Netherlands Society of Cardiology should make efforts to achieve a more uniform application of the guidelines in Dutch cardiovascular practice.

Most respondents thought that the physician can often decide for patients how risks and benefits should be weighed, and how quality of life should be weighed against life expectancy. It is, however, doubtful that a physician is actually good at assessing patient preferences in the context of prosthetic aortic valve selection. Physician perception of patient preferences in cardiovascular practice has not yet been investigated, but previous studies in the fields of vascular surgery and colorectal cancer screening have shown that physician perception of patient preferences may differ considerably from actual patient preferences [11, 12]. In fact, it was shown that although physicians usually think that they can adequately assess patient preferences, reality shows that they cannot do this. This is regardless of the clinical experience they have: from first-year residents to senior registrars, this deficit persists [13]. Because physicians have a major influence on patient decision-making, it is important for the physician to realise that their preferences may not be the preferences of the patient.

Patients who require aortic valve replacement need to be informed about the risks and benefits associated with the different prosthetic valve types in order to be able to participate in decision-making. The current study shows that in Dutch cardiovascular clinical practice major risks of the different therapeutic options are usually discussed with patients, and less common complications to a lesser extent. The observation that younger physicians more often report informing the patients regarding anticoagulation risks and that physicians working in an institution with a cardiac surgery program more often report informing patients about risks and benefits regarding a mechanical prosthesis, reflects the fact that evaluation of the risks and benefits is a complex process. This complexity is due to uncertainty about the various outcomes, difficulty to evaluate future events and the fact that most patients are unfamiliar with the medical consequences of their decisions [1].

The observation that both patient involvement and information provision to the patient are more common in institutions with a cardiac surgery program may be associated with the fact that these institutions have formal heart teams in which the cardiologists and surgeons discuss prosthetic valve selection.

To make patients more familiar with their treatment options it is important to inform patients, in a way that they can understand, about the benefits and risks associated with the different valve types. Well-informed patients are an essential requirement for successful shared decision-making. Only then can patients decide what is best for them and this will result in an optimisation of valve selection and an improved quality of life [14]. Of course, patients differ in their information needs. Some patients want to know every detail of their treatment, while other patients do not want to be involved at all. Furthermore, the educational level of the patient plays an important role in shared decision-making. It is known that incapacity to understand the process of medical decision-making is common [15]. Despite these differences in information needs and educational level, it is important that physicians should at least try to involve the patient. In some patients, however, this will require a lot of time and effort from the treating physician. Patient decision aids may be particularly useful in this setting. A decision aid will provide the patient with information about the disease, treatment options and risks and benefits that are associated with the different treatment options. Additionally, a decision aid helps the patient to place the provided information in his or her own context, considering the values and expectations of the patient. It is known that decision aids improve patient knowledge and lower decisional conflict without raising anxiety levels [16]. Only when patients are fully informed about their treatment options can they participate in the decision-making process and clarify their preferences to the physician. Although not all patients are willing to participate in decision-making, it is known that most patients do want to be informed [17] . Even patients who initially do not want to be involved in decision-making, do want to be involved once they are well informed [10, 18].

An important finding of the current study is the observed wide variation in physician preferences for a particular valve substitute and the association between medical speciality and prosthetic valve preferences. It may be that this variation is caused by differences in clinical practice setting, although we could not detect a difference between practices with or without an onsite cardiac surgery program. It may also be that physicians feel that in most patients both valve types are suitable. This is supported by the observation that in current practice there appears to be no difference in survival for adult patients with a mechanical or a bioprosthesis [19–22]. If the type of implanted prosthesis is not associated with patient survival, then the choice for a particular aortic valve prosthesis is mainly driven by valve-related event occurrence. Given the completely different nature of valve-related events between mechanical and bioprostheses, and subjective aspects of choosing between the hazards of bleeding due to anticoagulation (mechanical prostheses) and the hazards of re-operation (biological prostheses), informed patient preferences become very important.

In conclusion, this survey among Dutch cardiothoracic surgeons and cardiologists provides important information on current clinical decision-making regarding prosthetic aortic valve selection. Dutch cardiovascular professionals are of the opinion that prosthetic aortic valve selection should be done with the patient, and they usually convey most risks and benefits of the different options to the patient. Medical speciality influences both physician attitude with regard to prosthetic aortic valve selection and patient involvement, and preference for a particular valve substitute. The observed wide variation in prosthetic aortic valve preferences among Dutch cardiothoracic surgeons and cardiologists suggests that for most patients both mechanical and bioprosthetic valves are suitable, and that formal implementation of the concept of shared decision-making including the use of patient decision aids may be helpful for physicians and patients to improve patient information and patient participation in decision-making.
